# Longitudinal Learning Diagnosis: Minireview and Future Research Directions

**DOI:** 10.3389/fpsyg.2020.01185

**Published:** 2020-07-03

**Authors:** Peida Zhan

**Affiliations:** Department of Psychology, College of Teacher Education, Zhejiang Normal University, Jinhua, China

**Keywords:** longitudinal cognitive diagnosis, learning diagnosis, cognitive diagnostic assessment, assessment for learning, latent transition analysis

## Introduction

The basic premise of “teaching students according to their aptitude” is to have a relatively objective and accurate understanding of the students' current learning statuses (e.g., knowledge mastery level, learning motivation, learning attitude, and learning mode) and the developments/changes they undergo over time (e.g., did the students' knowledge mastery level improve, are the students' learning motivations enhanced). Measuring and improving individual development are topics that are actively tackled in psychological, educational, and behavioral studies.

In the past decades, learning diagnosis, which objectively quantifies students' current learning status and provides diagnostic feedback, has drawn increasing interest (Zhan, 2020). When focusing on fine-grained attributes (e.g., knowledge, skills, and cognitive processes), learning diagnosis can also be regarded as an application of cognitive diagnosis (Leighton and Gierl, 2007) in learning assessment. Although learning diagnosis aims to promote student learning based on diagnostic feedback and the corresponding remedial teaching (intervention), currently, only a few studies have focused on and evaluated the effectiveness of such feedback or remedial teaching (c.f., Tang and Zhan, submitted; Wu, 2019; Wang L. et al., in press; Wang S. et al., 2020). One of the main reasons is that cross-sectional design, which cannot measure individual growth in learning, is adopted by most current learning diagnoses. This issue may also be reflected in current learning diagnosis models (LDMs) or alternatively cognitive diagnosis models (for review, see Rupp et al., 2010; von Davier and Lee, 2019), which are the main tools for data analysis in learning diagnosis. Although various LDMs have been proposed and suggested by previous research, most of them are only applicable to cross-sectional data analysis, such as the deterministic inputs, noisy “and” gate (DINA) model (Junker and Sijtsma, 2001), the deterministic inputs, noisy “or” gate (DINO) model (Templin and Henson, 2006), the log-linear cognitive diagnosis model (LCDM) (Henson et al., 2009), and the generalized DINA (GDINA) model (de la Torre, 2011).

By contrast, longitudinal learning diagnosis evaluates students' knowledge and skills and identifies their strengths and weaknesses over a period of time. The data collected from longitudinal learning diagnosis provide researchers with the opportunities to develop models for learning tracking, which can be used to track individual growth over time as well as to evaluate the effectiveness of feedback. Compared to cross-sectional learning diagnosis, longitudinal learning diagnosis is more helpful when aiming to promote student learning.

Currently, longitudinal learning diagnosis is a new research direction that mainly stays in the model development stage and lacks practical applications and related topic research (e.g., missing data, measure invariance, and linking methods). Moreover, although some longitudinal LDMs have been proposed, these models still have some limitations that need to be further studied. Thus, for the rest of this opinion article, I will first make a minireview of current longitudinal LDMs and then I will elaborate on several future research directions that I believe are worth studying. With this opinion article, I hope to elicit more research attention toward longitudinal learning diagnosis.

## Minireview

To provide theoretical support for data analysis in longitudinal learning diagnosis, longitudinal LDMs are needed. However, the latent variables (namely, attributes) in LDMs are categorical (typically, binary). Therefore, the methods for modeling growth for continuous latent variables (e.g., longitudinal item response theory models) cannot be directly extended to capture growth in the mastery of attributes. For example, the change in the mastery of attributes cannot be directly modeled by the variance-covariance methods when assuming that multiple continuous latent variables follow a multivariate normal distribution (e.g., von Davier et al., 2011).

To this end, in recent years, several longitudinal LDMs have been proposed. They are summarized in Table 1. Current longitudinal LDMs can mainly be divided into two categories: the latent transition analysis (Collins and Wugalter, 1992)-based models (e.g., Li et al., 2016; Kaya and Leite, 2017; Chen et al., 2018a; Madison and Bradshaw, 2018; Wang et al., 2018a) and the higher-order latent structural (de la Torre and Douglas, 2004)-based models (e.g., Huang, 2017; Lee, 2017; Zhan et al., 2019a). The diagnostic results of these two model types have high consistency (Lee, 2017).

**Table 1:** Summary of longitudinal cognitive diagnosis models.

**Basic method**	**References**	**Basic model**	**Learning tracking**
Latent transition analysis (LTA)	Li et al., 2016	DINA	LTA with attribute-level transition probability matrix
	Kaya and Leite, 2017	DINA, DINO	LTA with attribute pattern-level transition probability matrix
	Madison and Bradshaw, 2018	LCDM	LTA with attribute pattern-level transition probability matrix
	Chen et al., 2018a	DINA	LTA with attribute pattern-level transition probability matrix
	Wang et al., 2018a	DINA	LTA with modeled attribute-level transition probabilities
Higher-order latent structural model	Lee, 2017	DINA	Latent growth curve model
	Huang, 2017	GDINA	Multilevel latent growth curve model
	Zhan et al., 2019a	Testlet-DINA, DINA	Variance-covariance method

*The article collection ended at April 11th 2020; listing only the first article of the proposed model; DINA, deterministic-inputs, noisy “and” gate model Junker and Sijtsma, 2001; DINO, deterministic-inputs, noisy “or” gate model Templin and Henson, 2006; LCDM, log-linear cognitive diagnosis model Henson et al., 2009; GDINA, generalized deterministic-inputs, noisy “and” gate model de la Torre, 2011; Testlet-DINA, deterministic-inputs, noisy “and” gate model for testlet design (see e.g., Zhan et al., 2019b)*.

The latent transition analysis-based methods estimate the transition probabilities from one latent class/attribute to another or the same latent class/attribute. Two main differences exist in these models. First, different measurement models were used. Reduced LDMs, e.g., the DINA model and the DINO model, were used by Li et al. (2016), Kaya and Leite (2017), Chen et al. (2018a), and Wang et al. (2018a), but a generalized LDM, i.e., the LCDM, was used by Madison and Bradshaw (2018). Second, the attribute-level transition probability matrix (i.e., attributes are transited independently from one other) was used by Li et al. (2016) and Wang et al. (2018a), but the attribute pattern-level transition probability matrix was used by Kaya and Leite (2017), Chen et al. (2018a), and Madison and Bradshaw (2018). In addition, different from Li et al. (2016), Kaya and Leite (2017), Chen et al. (2018a), and Madison and Bradshaw (2018), who directly estimated the transition probabilities, Wang et al. (2018a) used a set of covariates, such as a time-invariant general learning ability and intervention indicators, to model the transition probabilities. The effectiveness of different learning interventions was further considered by Zhang and Chang (2019). Additionally, to reduce modeling complexity, Chen et al. (2018a) and Wang et al. (2018a) assumed learning trajectories to be non-decreasing (i.e., respondents did not forget). However, this non-decreasing assumption may only be suitable for short-time interval assessments. Furthermore, by incorporating response times into LDMs (Wang et al., 2018a, 2019; Zhan et al., 2018a; Zhang and Wang, 2018) used response times to assist in measuring students' growth in attribute mastery.

Meanwhile, the higher-order latent structural model-based methods estimate the changes in a higher-order latent ability over time to further infer the changes of lower-order latent attributes. One of the representative models is the longitudinal higher-order DINA (Long-DINA) model (Zhan et al., 2019a), which is a multidimensional extension of the higher-order DINA model (de la Torre and Douglas, 2004). However, multidimensionality does not refer to different general abilities, but rather, the same general ability measured at different time points. As noted by Zhan et al. (2019a), the latent growth curve model instead of the variance-covariance method can also be employed in the third order. Lee (2017) proposed a growth curve DINA model, which can be seen as an alternative of the long-DINA model that incorporates the latent growth curve model but ignores the local item dependence among anchor or repeat items. Furthermore, Huang (2017) proposed a multilevel GDINA model for assessing growth, which can be seen as an extension of Lee's (2017) model in both measurement model part (i.e., from DINA model to GDINA model) and latent structural model part (i.e., from one-level growth curve model to multilevel growth curve model).

## Future Research Direction

Although the utility for analyzing the longitudinal learning diagnosis data of these longitudinal LDMs has been evaluated by some simulation studies and a few applications, these models are not without limitations, which need to be further studied. Based on current research on longitudinal learning diagnosis, I believe that the following are directions that are worthy of further study.

1. A systematic comparison between different longitudinal LDMs, which can provide theoretical suggestions for practitioners in choosing suitable models.
2. Only binary attributes (e.g., “1” means mastery and “0” means non-mastery) were considered in all current longitudinal LDMs. However, in actual teaching, it is challenging to use binary attributes to describe the growth of students, as they can be classified into only four categories between two adjacent time points, i.e., 0 → 0, 0 → 1, 1 → 0, and 1 → 1. Some small but existing growths are ignored, which in turn may lead students, especially those with low motivation to learn, to conclude that the current diagnostic feedback is ineffective or to abandon remedial action. Thus, further studies can attempt to extend the current models to handle polytomous attributes (Karelitz, 2004) and probabilistic attributes (Zhan et al., 2018b) because they can describe the learning growth in a more refined way than binary attributes.
3. Only outcome data (or item response accuracy) were considered in most current longitudinal LDMs. Although a few studies have incorporated item response time into current models (e.g., Wang et al., 2018b), future studies may attempt to introduce other types of process data (e.g., number of trial and error and operation process), or even biometric data (e.g., eye-tracking data; Man and Harring, 2019). Utilizing multimodal data can evaluate the growth of students in multiple aspects, which is conducive to a more comprehensive understanding of the development of students (Zhan, 2019).
4. All current longitudinal LDMs assumed that attributes are structurally independent, in that mastery of one attribute is not a prerequisite to the mastery of another. However, when attribute hierarchy (Leighton et al., 2004) exists, the development trajectory of students is not arbitrary and should be developed in such hierarchical order. Therefore, incorporating the attribute hierarchy into current longitudinal LDMs is worth trying.
5. A limited number of attributes at each time point were assumed in current longitudinal LDMs. In practice, a large number of attributes may involve more than 10 or 15 attributes at each time point. In such cases, using current longitudinal LDMs with existing parameter estimation algorithms may lead to unrobust parameter estimation. Thus, more powerful or efficient algorithms or special strategies may need to be introduced.
6. The simultaneity estimation strategy was adopted by almost all current longitudinal LDMs. This involves the reintegration of response data from multiple time points into one large response matrix, which is then analyzed as a whole (Zhan et al., 2019a). However, this strategy requires subjects to wait until all the tests end before an analysis of the results becomes available. Thus, using this strategy cannot provide timely diagnostic feedback to either students or teachers. In light of the foregoing, new estimation strategies for timely diagnostic feedback should be further studied (e.g., Zhan, 2020).
7. In addition to theoretical and methodological studies, the corresponding applied studies should also be strengthened. For example, a few studies have focused on and evaluated the effectiveness of diagnostic feedback or remedial teaching in promoting learning (cf. Tang and Zhan, submitted). Moreover, effective and systematic intervention methods based on longitudinal diagnostic feedback are also worth studying.
8. Adaptive learning system involving LDMs is also worthy of further study (e.g., Chen et al., 2018b; Tang et al., 2019). This system can diagnose an individual's latent attribute profile online while the assessment is being conducted.
9. Compared with cross-sectional learning diagnosis, the diagnostic accuracy and validity of longitudinal learning diagnosis used to depict the learning trajectories are more worthy of attention by researchers and practitioners. In addition to choosing a suitable longitudinal LDM, many factors such as the quality of the longitudinal test itself, the setting of a cognitive model, students' response attitude, cheating, and missing data will also affect the accuracy and validity of the diagnostic results. The impact of these factors on the longitudinal learning diagnosis and the corresponding compensation or detection methods are also worthy of further discussion.

Overall, there are still many issues related to longitudinal learning diagnosis that are worthy of discussion. In view of the advantages of longitudinal learning diagnosis compared with cross-sectional learning diagnosis, the former is more in line with the idea of assessment for learning (Wiliam, 2011) and the needs of formative assessments.

## Author Contributions

The author confirms being the sole contributor of this work and has approved it for publication.

## Conflict of Interest

The author declares that the research was conducted in the absence of any commercial or financial relationships that could be construed as a potential conflict of interest.
